# Auranofin, at clinically achievable dose, protects mice and prevents recurrence from *Clostridioides difficile* infection

**DOI:** 10.1038/s41598-020-64882-9

**Published:** 2020-05-07

**Authors:** Nader S. Abutaleb, Mohamed N. Seleem

**Affiliations:** 1 Department of Comparative Pathobiology, College of Veterinary Medicine, Purdue University, West Lafayette, IN 47907 USA; 2 Purdue Institute of Inflammation, Immunology, and Infectious Disease, West Lafayette, IN 47907 USA

**Keywords:** Drug discovery, Microbiology

## Abstract

*Clostridioides difficile* is the leading cause of nosocomial infections and a worldwide urgent public health threat. Without doubt, there is an urgent need for new effective anticlostridial agents due to the increasing incidence and severity of *C. difficile* infection (CDI). The aim of the present study is to investigate the *in vivo* efficacy of auranofin (rheumatoid arthritis FDA-approved drug) in a CDI mouse model and establish an adequate dosage for treatment. The effects of increased *C. difficile* inoculum, and pre-exposure to simulated gastric intestinal fluid (SGF) and simulated intestinal fluid (SIF), on the antibacterial activity of auranofin were investigated. Auranofin’s *in vitro* antibacterial activity was stable in the presence of high bacterial inoculum size compared to vancomycin and fidaxomicin. Moreover, it maintained its anti-*C. difficile* activity after being exposed to SGF and SIF. Upon testing in a CDI mouse model, auranofin at low clinically achievable doses (0.125 mg/kg and 0.25 mg/kg) significantly protected mice against CDI with 100% and 80% survival, respectively. Most importantly, auranofin (0.125 mg/kg and 0.25 mg/kg) significantly prevented CDI recurrence when compared with vancomycin. Collectively, these results indicate that auranofin could potentially provide an effective, safe and quick supplement to the current approaches for treating CDI.

## Introduction

*Clostridioides difficile* is the worldwide leading cause of nosocomial infections and antibiotic-associated diarrhea^1^. A recent report released by the Centers for Disease Control and Prevention (CDC) stated that about 223,900 patients were hospitalized with *C. difficile* infections (CDI) in the United States in 2017, which was associated with around 12,800 mortality cases and in excess of $1 billion healthcare cost^2^.

CDI symptoms range from mild to severe watery diarrhea to more severe life threatening complications such as pseudomembranous colitis, toxic megacolon, colon perforation, sepsis, systemic inflammatory response syndrome and shock^3^. Disease manifestations are attributed to the toxin-mediated damage elicited by the two major toxins TcdA and TcdB. These toxins catalyze inactivation of host GTPases (Rac, Rho and CDC42) and perturbation of actin cytoskeleton, ultimately causing intense inflammation, loss of tight junctions of the intestinal mucosal layer, enormous fluid secretion, cell rounding and finally necrosis and apoptosis of the colonic mucosal cells^4,5^. The incidence and severity of CDI has increased dramatically due to the overuse of antibiotics and the emergence of hypervirulent epidemic strains such as, but not limited to, pulsed-field gel type North American pulsotype 1 (NAP1) or PCR ribotype 027, which were responsible for several outbreaks globally^6,7^. Moreover, the clinical management of CDI is hindered by the ability of *C. difficile* to produce spores which are highly resistant to environmental conditions, antibiotics and disinfection processes. Spores can persist on unsuitable environments for long periods and spread in the environment^8^. Once ingested by susceptible hosts, these spores germinate, in response to bile acids in the gut, into vegetative cells that colonize in the intestine, produce toxins and establish infection^9^. Consequently, *C. difficile* spores serve as the major cause CDI dissemination and recurrence.

Even though the overuse of antibiotics is responsible for CDI, the management of CDI requires antibiotic administration. Currently, only two drugs are approved for treatment of both non-severe and severe CDI; vancomycin and fidaxomicin. While metronidazole is not FDA-approved for treatment of CDI, it was previously recommended as a first-line therapeutic option for CDI in adults. It use is now restricted to non-severe CDI cases when patients are unable to obtain or be treated with vancomycin or fidaxomicin^10^. Vancomycin or metronidazole treatments are limited by the high treatment failure (22% with metronidazole, and 14% with vancomycin), and the high recurrence rate (25–30%)^7,11^. Furthermore, fidaxomicin has lower recurrence rate due to its less disturbance effect on gut microbiota; yet, its high cost restricts its use^12–14^. Further compounding the CDI problem is the emerging resistance or reduced susceptibility to these antibiotics^13,15^. Thus, the critical and the unmet need for developing new anti-CDI therapeutics cannot be overemphasized.

Auranofin is an FDA-approved anti- rheumatoid arthritis drug, with a well-studied safety profile for human use^16,17^. Recently, auranofin has gained interest in repurposing for treatment of bacterial and parasitic infections^18–23^. Furthermore, it is undergoing Phase II clinical trials for the treatment of amoebic dysentery, giardiasis (NCT02736968) and tuberculosis (NCT02968927). Auranofin possesses strong antibacterial and antifungal activities^17,22–25^. We previously demonstrated that auranofin has a potent anticlostridial activity with strong inhibition of both toxins and spores production *in vitro*^24^. We hypothesized that auranofin’s potent antibacterial and antivirulence activity against *C. difficile* would be beneficial in treating mice infected with *C. difficile* in an *in vivo* CDI mouse model. The main objective of the present study was to investigate the *in vivo* efficacy of auranofin treatment in a CDI mouse model and to study the ability of auranofin to prevent CDI recurrence. In addition, this study established the doses needed to achieve 100% protection in CDI mouse model and prevent recurrence. The impact of increasing *C. difficile* inoculum, and its pre-exposure to simulated gastric fluid and simulated intestinal fluid, on the antibacterial activity of auranofin were also investigated.

## Results and Discussion

### The effect of *C. difficile* inoculum size on the antibacterial activity of auranofin

*C. difficile* is known to colonize the intestinal tract in large populations. Additionally, a higher inoculum (~10^6^ CFU/mL) is often used to infect animals in *in vivo* CDI models. It was reported that the bacterial burden recovered from cecal and fecal contents of infected mice had averages of ~10^6^ to 10^7^ CFU/g^26–28^. The dependence of the antibacterial activity of anticlostridial drugs on the inoculum effect is an important consideration, especially for a weakly absorbed drug like auranofin (85% of the administered dose is not absorbed and recovered in feces)^29^. After being administered orally, auranofin will be localized in the gut, and target the colonizing *C. difficile* populations. However, the standard antibacterial susceptibility assays typically evaluate test agents at a lower inoculum size (~10^5^ CFU/mL). Thus, we evaluated the impact of the high *C. difficile* inoculum (HI, ~5 × 10^7^ CFU/mL), compared with the standard inoculum (SI, 5 × 10^5^ CFU/mL), on the antibacterial activity of auranofin. Upon testing against SI, auranofin exhibited a potent *in vitro* activity against the *C. difficile* strains tested with MIC values ranging from 0.25–1 µg/mL (Table 1), in agreement with a previous study^24^. Furthermore, auranofin’s antibacterial activity was identical to or one-fold higher, as the inoculum size increased from 10^5^ CFU/mL to 10^7^ CFU/mL (Table 1), suggesting that its activity was not impacted by increasing the inoculum size. Its MIC_90_ was not affected by the increase in the inoculum size. Fidaxomicin MICs, in agreement with a previous study^30^ were not affected by increasing the *C. difficile* inoculum size (MICs of HI were equal to or one-fold higher than SI MICs except the MICs against *C. difficile* NR-49278 that increased by three-fold). Additionally, its MIC_90_ with the HI was the same as that of the SI. Conversely, vancomycin’s activity was negatively impacted by the increased inoculum size (MIC increased three-fold against *C. difficile* NR-49278, *C. difficile* NR-49281, and *C. difficile* NR-49284), in accordance with a previous report^30^. Additionally, its MIC_90_ with the HI was one-fold higher than that of the SI.

**Table 1:** MICs (µg/mL) of auranofin and control antibiotics against *C. difficile* clinical isolates at standard and high inocula.

*C. difficile* strains	Auranofin		Vancomycin		Fidaxomicin	
	SI	HI	SI	HI	SI	HI
ATCC BAA 1870	1	1	1	1	0.03	0.03
ATCC 43255	0.5	0.5	1	2	0.015	0.015
NR-49277	0.5	0.5	1	2	0.03	0.03
NR-49278	0.25	0.25	0.25	1	0.007	0.007
NR-49281	0.25	0.25	0.25	1	0.007	0.015
NR-49284	0.25	0.25	0.25	1	0.015	0.015
NR-49285	0.5	0.5	0.5	1	0.015	0.03
NR-49286	0.25	0.25	0.25	0.25	0.007	0.007
NR-49288	0.25	0.5	0.5	1	0.007	0.03
NR-49290	0.25	0.25	0.5	1	0.015	0.015
MIC_90_	**0.5**	**0.5**	**1**	**2**	**0.03**	**0.03**

SI, standard inoculum (∼5 × 10^5^ CFU/mL); HI, high inoculum (∼5 × 10^7^ CFU/mL); MIC_90_, the concentration of the test agent that inhibited the growth of 90% of the tested strains.

### The effect of simulated gastric fluid (SGF) and simulated intestinal fluid (SIF) on the antibacterial activity of auranofin

It is important to analyze the stability of drugs, especially those intended for oral administration, in harsh conditions of the gastrointestinal tract (GIT). The stability of a drug in gastric and intestinal fluids provides evidence whether it is prone to degradation process by the effect of GIT fluids prior to absorption^31^. Drugs stability in presence of the GIT fluids can be investigated by incubating the drug in simulated gastric fluid (for 1-2 hours) and simulated intestinal fluids (for 3-4 hours) to mimic the *in vivo* drug exposure to these fluids^31,32^. To investigate the effect of SGF and SIF on the antibacterial activity of auranofin against *C. difficile*, auranofin, and vancomycin and fidaxomicin (control antibiotic) were incubated with SGF and SIF for 2, 4 and 24 hours and their MICs against 2 clinical *C. difficile* strains were determined. As depicted in Table 2A, after incubation with SGF, the MICs of auranofin did not increase against *C. difficile* ATCC BAA 1870, even after 24 hours exposure, and increased by one-fold only against *C. difficile* ATCC 43255 after 24 hours exposure. This result suggests that auranofin was stable after exposure to the gastric pH and was not affected by the enzymes of gastric fluids. Similarly, vancomycin and fidaxomicin MICs (after exposure to SGF) were similar to or one-fold higher than their corresponding MICs in absence of SGF. Furthermore, auranofin MICs, after incubation with SIF up to 24 hours, were equal to or one-fold higher than its MIC without incubation with SIF (Table 2B), suggesting that auranofin was not affected by exposure to the intestinal fluids. The antibacterial activity of vancomycin and fidaxomicin also, were not affected by incubation with SIF (MICs are equal to or one-fold higher than their corresponding MICs without exposure to SIF) (Table 2B). This result came in coincidence with a previous report^30^.

**Table 2:** MICs (µg/mL) of auranofin and control antibiotics against *C. difficile* clinical isolates after incubation with: (**A)** simulated gastric fluid (SGF), (**B)** simulated intestinal fluid (SIF), for the corresponding times (hours).

(A) Simulated gastric fluid (SGF)												
*C. difficile* strains	**Auranofin**				**Vancomycin**				**Fidaxomicin**			
	**0 h**	**2 h**	**4 h**	**24 h**	**0 h**	**2 h**	**4 h**	**24 h**	**0 h**	**2 h**	**4 h**	**24 h**
ATCC BAA 1870	1	1	1	1	1	1	1	2	0.03	0.03	0.03	0.03
ATCC 43255	0.5	0.5	0.5	1	1	1	1	1	0.015	0.015	0.015	0.015
**(B) Simulated intestinal fluid (SIF)**												
***C. difficile*** **strains**	**Auranofin**				**Vancomycin**				**Fidaxomicin**			
	**0 h**	**2 h**	**4 h**	**24 h**	**0 h**	**2 h**	**4 h**	**24 h**	**0 h**	**2 h**	**4 h**	**24 h**
ATCC BAA 1870	1	1	1	2	1	1	1	2	0.03	0.03	0.03	0.06
ATCC 43255	0.5	1	1	1	1	1	1	2	0.015	0.015	0.015	0.03

### *In vivo* efficacy of auranofin in a CDI mouse model

**Figure 1:**
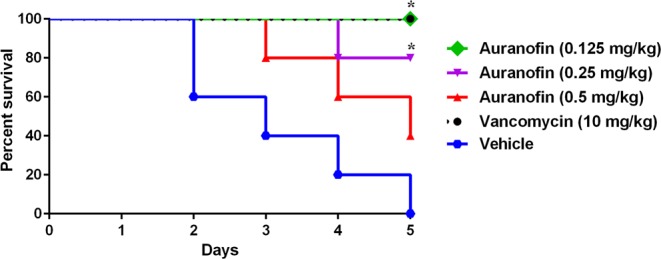
Auranofin protects mice against CDI. Mice were treated with auranofin (0.125 mg/kg, 0.25 mg/kg, and 0.5 mg/kg), vancomycin (10 mg/kg), or the vehicle for 5 days after infection with *C. difficile* spores. Kaplan–Meier survival curves were analyzed using a log-rank (Mantel–Cox) test. Asterisks (*) denote statistical significant difference between mice treated with either auranofin, or vancomycin in comparison with the vehicle-treated mice.

**Figure 2:**
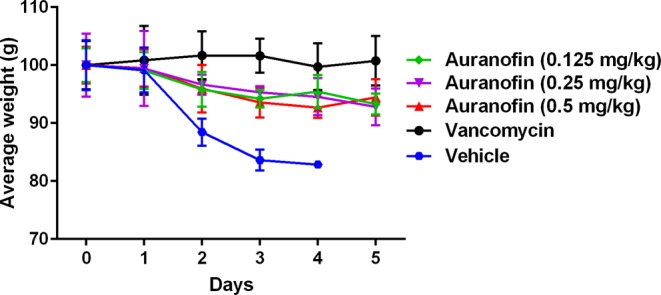
Average relative weight of all surviving mice. Infected mice were treated with auranofin (0.125 mg/kg, 0.25 mg/kg, and 0.5 mg/kg), vancomycin (10 mg/kg), or the vehicle for 5 days and weighed daily till the end of the experiment. The data are presented as percent relative weight (mean ± standard deviation) for each group.

The potent antibacterial and antivirulent activities of auranofin against *C. difficile*^24^ in addition to its stability in SGF and SIF prompted us to investigate its efficacy in a CDI mouse model and its potential to protect mice from CDI recurrence. In our study, CDI was established first before treatment. Three groups of mice were treated with (0.125 mg/kg, 0.25 mg/kg and 0.5 mg/kg) of auranofin. Two additional groups were used, positive control (vancomycin) and negative control (vehicle) groups. Mice were treated with the corresponding drugs for 5 days and monitored for disease symptoms. As shown in Fig. 1, vancomycin (10 mg/kg) protected 100% of mice up to 5 days, as previously reported^27,33^. In addition, auranofin, at low clinically achievable concentration (0.125 mg/kg), was able to protect 100% of the mice against *C. difficile* during the 5-days treatment period. Interestingly, the higher doses (0.25 mg/kg and 0.5 mg/kg) protected only 80% and 40% of mice, respectively. There was no significant difference in survival between vancomycin-treated, and auranofin (0.25 mg/kg)- treated groups. This effect (lower dose more effective) could be due to the potent anticommensal activity of auranofin against the human gut intestinal microbiota^34^, which could increase by increasing the administered dose leading to establishment of *C. difficile* colonization in the intestine and higher mortality. This activity was also, reported for niclosamide against *C. difficile* where the lower dose (2 mg/kg) protected mice from CDI more effectively than higher doses (10 mg/kg and 50 mg/kg)^35^. The effectiveness of lower dose of auranofin could also be explained by their potent anti-inflammatory activity. It was reported that the anti-inflammatory drug, indomethacin, increased the severity of *C. difficile* infection in mice^36^. Additionally, in previous study investigating the efficacy of auranofin against vancomycin-resistant enterococci peritonitis, lower doses of auranofin provided the best protection (100%)^37^. Moreover, Fig. 2 depicts the mean relative daily weight for all mice groups. The control group (vehicle-treated) showed weight loss starting day 2 after infection and their weight continued to decrease till day 4. Conversely, vancomycin-treated mice did not show weight loss till day 5. Similarly, auranofin-treated mice maintained a stable body weight with a minor weight reduction till day 5 (Fig. 2).

**Figure 3:**
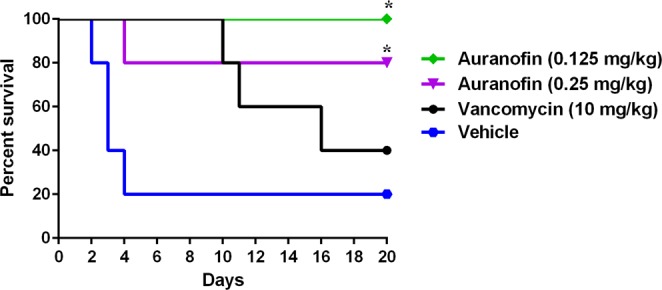
Efficacy of auranofin against CDI recurrence. Mice were treated with auranofin (0.125 mg/kg, and 0.25 mg/kg), vancomycin (10 mg/kg), or the vehicle for 5 days after infection with *C. difficile* spores and the treatments were stopped afterwards. Mice were monitored for survival. Kaplan–Meier survival curves were analyzed using a log-rank (Mantel–Cox) test. Asterisks (*) denote statistical significant difference between mice treated with either auranofin, or vancomycin in comparison with vehicle-treated mice.

**Figure 4:**
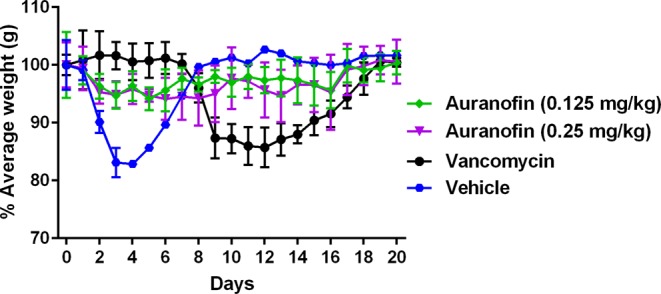
Average relative weight of all surviving mice in *C. difficile* recurrence experiment. Infected mice were treated with auranofin (0.125 mg/kg and 0.25 mg/kg), vancomycin (10 mg/kg), or the vehicle for 5 days and treatments were stopped thereafter. Mice were weighed daily till the end of the experiment. The data are presented as percent relative weight (mean ± standard deviation) for each group.

Symptomatic recurrence of CDI occurs in approximately 20% of patients and is challenging to treat^38–42^. In addition to subsequent prolongation of *C. difficile* shedding and transmission, 1 out of every 5 patients experienced *C. difficile* recurrence episode died within 30 days of diagnosis^43^. Then, we sought to investigate this promising activity of auranofin in preventing *C. difficile* recurrence. Mice were infected and treated for 5 days and mice were monitored for survival and possible *C. difficile* recurrence until the 20^th^ day. Vancomycin-treated mice, in accordance with a previous study^33^, were susceptible to *C. difficile* recurrence where 60% of mice died after stopping vancomycin treatment. In contrast, auranofin (0.125 mg/kg and 0.25 mg/kg), significantly protected mice from CDI recurrence with 100% and 80% survival, respectively after 20 days (Fig. 3). Additionally, the relative body weight results (Fig. 4) showed that vehicle-treated group started to lose weight on day 2 and the weight loss continued till day 4. Afterwards, the surviving mice showed clinical recovery and started to gain weight till they returned to the normal weight. Vancomycin-treated mice, in coincidence with a previous report^33^, maintained their weight till the start of recurrence after the treatment discontinuation. By day 9, mice started to lose weight which continued to decrease until day 12. Thereafter, the average weight of surviving mice (40%) started to increase till they reached the normal weight. In contrast, auranofin-treated (0.125 mg/kg and 0.25 mg/kg) groups maintained a stable body weight along the duration of the experiment.

A point worth noting, auranofin doses used in this study are achievable clinically. The recommended long term dosing regimen of auranofin in adult patients is 6-9 mg daily, and 0.1–0.25 mg/kg/day for children, in a single dose or divided doses^44,45^. Consequently, the most effective dose in this study, (0.125 mg/kg), is within range of doses administered clinically to humans. In addition, the therapeutic benefits and toxicity profile of auranofin have been monitored in clinical trials in more than 5,000 rheumatoid arthritis patients taking the drug and some of whom were monitored for more than 7 years. Auranofin did not show any evidence of cumulative toxicity and it was approved by the FDA for long-term treatment of rheumatoid arthritis in 1985^46^. Furthermore, auranofin is approved for long-term treatment of rheumatoid arthritis, a much greater course than would be expected for anticlostridial therapeutics.

In conclusion, this study investigated the effectiveness of auranofin, at clinically achievable doses, as a CDI therapeutic. Auranofin’s *in vitro* antibacterial activity was stable in the presence of high bacterial inoculum size compared to vancomycin and fidaxomicin. Moreover, it maintained its anti-*C. difficile* activity after being exposed to SGF and SIF. Interestingly, it significantly protected mice against CDI at low doses (0.125 mg/kg and 0.25 mg/kg). Most importantly, auranofin (0.125 mg/kg and 0.25 mg/kg) significantly prevented CDI recurrence. These results indicate that auranofin warrants further investigation as a new CDI treatment option.

## Materials and Methods

### Bacterial strains, media and reagents

All experiments were performed following the relevant guidelines and regulations of the Purdue University Institutional Biosafety Committee. *C. difficile* strains (Table 3) were obtained from the Biodefense and Emerging Infections Research Resources Repository (BEI Resources) (Manassas, VA, USA), and the American Type Culture Collection (ATCC) (Manassas, VA, USA). Brain heart infusion broth was purchased from Becton, Dickinson and Company (Cockeysville, MD, USA). Hemin and vitamin K were obtained from Sigma-Aldrich (Saint Louis, MO, USA). Yeast extract, sucrose and L-cysteine were purchased from Fisher Scientific (Fail Lawn, NJ, USA). Phosphate buffered saline (PBS) (Corning, Manassas, VA, USA), pepsin from porcine gastric mucosa, pancreatin from porcine pancreas, hydrochloric acid (HCl), sodium chloride (NaCl), sodium hydroxide (NaOH), bovine serum albumin (Sigma-Aldrich, Saint Louis, MO, USA), monobasic potassium phosphate (KH_2_PO_4_) (Macron chemicals, Center Valley, PA, USA), vancomycin hydrochloride, gentamicin sulfate, kanamycin monosulfate, taurocholic acid (Chem-Impex, Wood Dale, IL, USA), metronidazole (Alfa Aesar, Ward Hill, MA, USA), and colistin sulfate, fidaxomicin (Cayman Chemical, Ann Arbor, MI, USA) were purchased commercially.

**Table 3:** *C. difficile* strains used in this study.

*C. difficile* Strains	Source/Description
ATCC BAA-1870 (4118)	*tcdA*^a^*, tcdB*^b^ and CDT^c^ genes. Ribotype 027 and NAP^d^.
ATCC 43255 (*VPI 10463)*	Abdominal wound. *tcdA* and *tcdB*, ribotype 087.
NR-49277 (20100502)	Stool sample, Colorado, 2010. *tcdA*, *tcdB*, *tcdC*^*e*^, and CDT. Ribotype 019, NAP1.
NR-49278 (20100207)	Stool sample, New York, 2010. *tcdA*, *tcdB*, *tcdC* and CDT. Ribotype 027, NAP1.
NR-49281 (20110052)	Stool sample, northeastern USA, 2010. *tcdA*, *tcdB*, *tcdC* and CDT. Ribotype 027, NAP1.
NR-49284 (20120015)	Stool sample, New York, USA, 2011. *tcdA*, *tcdB*, *tcdC* and CDT. Ribotype 027, NAP1.
NR-49285 (20110979)	Stool sample, midwestern USA, 2011. *tcdA*, *tcdB*, *tcdC* and CDT. Ribotype 027, NAP1.
NR-49286 (20110999)	Stool sample, western/midwestern USA, 2011. *tcdA*, *tcdB*, *tcdC* and CDT. Rribotype 027, NAP1.
NR-49288 (20110870)	Stool sample, Tennessee, USA, 2011. *tcdA*, *tcdB*, *tcdC* and CDT. Ribotype 027, NAP1.
NR-49290 (20120187)	Stool sample, Tennessee, USA, 2011. *tcdA*, *tcdB*, *tcdC* and CDT. Ribotype 019, NAP1.

^a^*tcdA*, toxin A gene; ^b^*tcdB*, toxin B gene; ^c^CDT, binary toxin; ^d^NAP, North American pulsed-field gel electrophoresis type; ^e^*tcdC*, Anti-sigma factor gene.

### Evaluation of the effect of *C. difficile* inoculum size on the antibacterial activity of auranofin

The broth microdilution assay was used to determine the impact of *C. difficile* inoculum size on the minimum inhibitory concentrations (MICs) of auranofin and control antibiotics, as described previously^24,47,48^. Briefly, standard inoculum (SI: ~5 × 10^5^ CFU/mL) and high inoculum (HI: ~5 × 10^7^ CFU/mL) of each *C. difficile* strain were prepared in brain heart infusion supplemented broth (BHIS) and tested against auranofin and control antibiotics. Plates were then, incubated anaerobically at 37 °C for 48 hours. MICs reported are the lowest drug concentration that completely suppressed the growth of bacteria, as observed visually.

### Activity of auranofin after exposure to simulated gastric fluid (SGF) and simulated intestinal fluid (SIF)

Simulated gastric fluid (SGF) and simulated intestinal fluid (SIF) were prepared as described earlier^32,49^. Briefly, SGF (pH = 1.2) was prepared by dissolving NaCl (2 g) and pepsin (3.2 g) in 7 mL of concentrated HCl and deionized water was subsequently added to make up a final volume of 1 L. Then, the pH was adjusted to 1.2. To prepare SIF (pH = 6.8), 6.8 g of KH_2_PO_4_ was dissolved in 250 mL of water, and 77 mL of 0.2 N NaOH and 500 mL of deionized water were added. Afterwards, 10 g of pancreatin was added, and the pH of the resulting solution was adjusted to 6.8.

The broth microdilution assay^24,47,48^ was used to determine the MICs of auranofin and control antibiotics in presence of SGF and SIF. Briefly, auranofin and control drugs were incubated with each of SGF and SIF for 2, 4 and 24 hours. After the corresponding times, broth microdilution assay was performed to determine the MICs of the tested drugs.

### Preparation of *C. difficile* spores for mice infection

*C. difficile* spores were prepared as described earlier^50^. Briefly, *C. difficile* ATCC 43255 was inoculated onto BHIS agar and incubated anaerobically for 5 days. Spores were collected anaerobically using PBS containing 10% bovine serum albumin, heated at 70 °C for 20 minutes to get rid of vegetative cells and counted by dilution and plating onto BHIS supplemented with 0.1% taurocholic acid. Spores were then, stored at 4°C overnight before infecting mice.

### *In vivo* efficacy of auranofin in a CDI mouse model

#### CDI mouse model

The study was reviewed, approved and performed following the guidelines of the Purdue University Animal Care and Use Committee (PACUC) and according to the recommendations in the Guide for the Care and Use of Laboratory Animals of the National Institutes of Health. Mice were housed in individually ventilated autoclaved cages and received sterile food and water ad libitum throughout the duration of the experiment. CDI mouse model was performed as described previously^33^ with modifications. Since disruption of microbiota depends on mice drinking naturally, we performed three modifications: (1) increasing the concentrations of antibiotics to ensure microbiota disruption, (2) adding 7.5% sucrose to the drinking water containing antibiotics to overcome the very bitter taste of the antibiotics in drinking water, as mice are expected to decrease their rate of water consumption due to its bitter taste, and (3) extending the duration of administering antibiotic cocktail in drinking water to 5 days to ensure microbiota disruption. Eight-week-old female pathogen-free C57BL/6 mice (Jackson, ME, USA) were pre-treated with an antibiotic cocktail in sterile drinking water to disrupt the mice normal intestinal microflora, reducing the colonization resistance and facilitating infection with the toxigenic strain of *C. difficile*. The cocktail contained kanamycin (1.2 mg/mL), gentamicin (0.105 mg/mL), colistin (2550 U/mL), metronidazole (0.645 mg/mL), vancomycin (0.135 mg/mL) and sucrose (75 mg/mL) for 5 days. Afterwards, mice were switched to regular autoclaved water for 2 days and they received a single dose of clindamycin (10 mg/kg) intraperitoneally 1 day prior to *C. difficile* challenge.

For infection, mice were restrained and infected intragastrically with 1.3 ×10^6^ spores of *C. difficile* ATCC 43255 via oral gavage using a ball tipped metal feeder. Number of spores used were re-counted after infection to confirm the infected dose.

#### *In vivo* efficacy of different doses of auranofin in a CDI mouse model

Following infection, mice were randomly allocated into groups (n = 5) for treatment. Two hours post-infection, three groups were treated orally with auranofin (0.125 mg/kg, 0.25 mg/kg and 0.5 mg/kg), one group was treated with vancomycin (10 mg/kg) via oral gavage, and one group was treated orally with the vehicle (10% DMSO in PBS). Treatments were continued once daily for five days and mice were checked (6 times daily) for disease signs (including weight loss, behavioral changes, hunched posture, decreased activity, wet tail and diarrhea).

#### *In vivo* efficacy of auranofin in *C. difficile* recurrence

In order to investigate the activity of auranofin in preventing *C. difficile* recurrence, mice were infected, as described above and two groups were treated orally with auranofin (0.125 mg/kg and 0.25 mg/kg), one group was treated with vancomycin (10 mg/kg) via oral gavage, and one group was treated orally with the vehicle (10% DMSO in PBS) for 5 days. Treatments were stopped after 5 days and mice were monitored (6 times daily) for disease signs and recurrence of infection till the 20^th^ day. Then, mice were humanely euthanized at 21^st^ day post-infection using CO_2_ asphyxiation.

### Statistical analyses

The survival data were analyzed by Log-rank (Mantel-Cox) test utilizing GraphPad Prism version 6.00 for Windows (GraphPad Software, La Jolla, CA, USA).

### Ethical approval

All animal housing and experiments were reviewed, approved and performed under the guidelines of the Purdue University Animal Care and Use Committee and carried out in strict accordance with the recommendations in the Guide for the Care and Use of Laboratory Animals of the National Institutes of Health.

## Data Availability

Data presented in this study are available from the corresponding author upon a proper request.
